# The Non-Homologous End Joining Protein PAXX Acts to Restrict HSV-1 Infection

**DOI:** 10.3390/v9110342

**Published:** 2017-11-16

**Authors:** Ben J. Trigg, Katharina B. Lauer, Paula Fernandes dos Santos, Heather Coleman, Gabriel Balmus, Daniel S. Mansur, Brian J. Ferguson

**Affiliations:** 1 Department of Pathology, University of Cambridge, Tennis Court Road, Cambridge CB2 1QP, UK; bjt40@cam.ac.uk (B.J.T.); kbl23@cam.ac.uk (K.B.L.); hmc@mole.bio.cam.ac.uk (H.C.); 2 Laboratory of Immunobiology, Department of Microbiology, Immunology and Parasitology, Universidade Federal de Santa Catarina, Santa Catarina 88040-900, Brazil; fernandes.santos.paula@gmail.com (P.F.d.S.); daniel.mansur@ufsc.br (D.S.M.); 3 Wellcome Trust/Cancer Research UK Gurdon Institute, University of Cambridge, Cambridge CB2 1QN, UK; gb318@cam.ac.uk; 4 Wellcome Trust Sanger Institute, Cambridge CB10 1HH, UK

**Keywords:** herpes simplex virus 1, HSV-1, DNA damage response, DDR, PAXX, c-NHEJ, classical non-homologous end joining

## Abstract

Herpes simplex virus 1 (HSV-1) has extensive interactions with the host DNA damage response (DDR) machinery that can be either detrimental or beneficial to the virus. Proteins in the homologous recombination pathway are known to be required for efficient replication of the viral genome, while different members of the classical non-homologous end-joining (c-NHEJ) pathway have opposing effects on HSV-1 infection. Here, we have investigated the role of the recently-discovered c-NHEJ component, PAXX (Paralogue of XRCC4 and XLF), which we found to be excluded from the nucleus during HSV-1 infection. We have established that cells lacking PAXX have an intact innate immune response to HSV-1 but show a defect in viral genome replication efficiency. Counterintuitively, *PAXX*^−/−^ cells were able to produce greater numbers of infectious virions, indicating that PAXX acts to restrict HSV-1 infection in a manner that is different from other c-NHEJ factors.

## 1. Introduction

**Figure 1:**
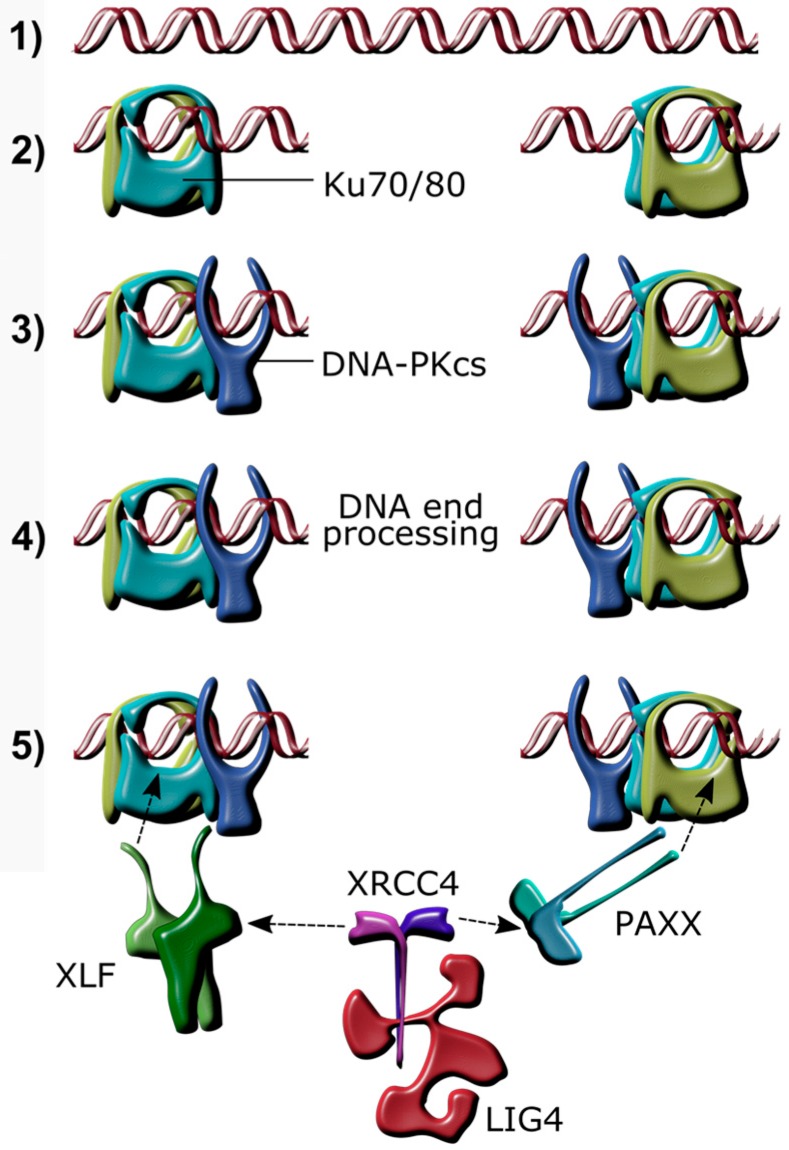
Schematic overview of non-homologous end joining after a DNA double strand break. (**1**) Linear DNA; (**2**) A double strand break (DSB) caused by genotoxic damage is bound by the Ku70/80 heterodimer, which slides along the DNA to make room for subsequent complex formation; (**3**) DNA-dependent protein kinase, catalytic subunit (DNA-PKcs) binds to the Ku70/80 heterodimer; (**4**) Factors, including aprataxin, Aprataxin And PNKP Like Factor (APLF), Polynucleotide Kinase 3′-Phosphatase (PNKP), Artemis, Werner Syndrome RecQ Like Helicase (WRN), DNA polymerase lambda (Polλ), and DNA polymerase mu (Polμ) modify the ends of the DNA if necessary; (**5**) Paralogue of XRCC4 and XLF (PAXX) and/or XRCC4-like factor (XLF) bind Ku70/80 and recruit the XRCC4/Ligase 4 (LIG4) complex, which in turn ligates the DNA ends together.

Mammalian cells have evolved mechanisms to detect and repair damaged DNA, in order to maintain genomic integrity. Causes of DNA damage are diverse and include ionizing radiation, intracellular reactive oxygen species, malfunction of endogenous processes, infections, and chemotherapeutic drugs. The most toxic DNA lesions for a cell are DNA double strand breaks (DSB). Non-homologous end joining (NHEJ) is a key process for DSB repair (Figure 1), particularly when homologous recombination (HR), which requires the presence of an identical DNA sequence for repair, is not possible [1]. The rejoining of DNA DSBs by NHEJ is initiated when free DNA ends are bound by the Ku70/80 heterodimer, which acts as a molecular scaffold and recruits the DNA-dependent protein kinase catalytic subunit (DNA-PKcs) [2,3,4]. X-ray cross complementing protein (XRCC4), XRCC4-like factor (XLF), and paralogue of XRCC4 and XLF (PAXX) are also recruited to the Ku heterodimer [5,6,7,8,9]. The ligation of the broken DNA ends is performed by DNA ligase IV (LIG4), which is activated through binding to XRCC4 and XLF [5,10]. PAXX is the most recently identified component of the classical NHEJ (c-NHEJ) complex, and was found due to its high degree of structural similarity with XRCC4 and XLF [6,7,8]. PAXX does not bind directly to DNA, but binds to DNA-bound Ku80 to stabilise the NHEJ-complex assembly [6,11,12].

While being essential for the rapid, template-independent repair of DSBs, components of the c-NHEJ machinery have other cellular functions. For instance, DNA damage response (DDR) proteins, including those involved in NHEJ, are also involved in the sensing of intracellular foreign DNA that results in the initiation of an innate immune response by triggering Type 1 interferon (IFN-I) production [13]. These include several cytoplasmic and nuclear DNA sensors, such as interferon γ-inducible protein 1 (IFI16) [14,15], DNA-PKcs [16,17], Ku70/80 [18,19], Double-strand break repair protein MRE11 (MRE11) [20], and RAD50 [21], which can activate a variety of innate immune signalling outputs [22,23] but mostly lead to IFNβ induction by activation of the Stimulator of interferon genes-Tank-binding kinase-Interferon Regulatory Factor 3 (STING-TBK-IRF3) pathway [14,24]. In response, herpes simplex virus type 1 (HSV-1) has evolved to inhibit these pathways, providing a partial explanation for the presence of multiple DNA sensing mechanisms [25,26,27].

The nuclear-replicating HSV-1 has extensive interactions with the DNA repair machinery, partly since its dsDNA genome contains gaps, nicks, and DNA-ends that pose targets for cellular DDR and innate immune DNA sensing mechanisms [28]. The activation of these pathways can mount a hostile cell-intrinsic antiviral response, for example through silencing of viral gene expression, cell cycle arrest, and apoptosis [29,30]. One of the earliest studies investigating the interaction of HSV-1 with a component of the NHEJ complex showed that HSV-1 attenuates the activity of DNA-PKcs via the viral immediate-early protein ICP0 [31]. This study indicated that DNA-PKcs has antiviral effects and, although the consequences of this interaction between DNA-PKcs and ICP0 are not fully resolved, other NHEJ proteins may be involved in this interaction [32]. These findings are coherent with a recent study, demonstrating that HSV-1 infection of cortical rat neurons downregulates the expression level of Ku80, via proteasomal degradation, leading to aberrant DDR processes in these cells [33].

However, DDR components can also promote HSV-1 infection. HSV-1 DNA synthesis occurs in the nucleus in distinct replication compartments. It has been proposed that viral replication follows an intricate rolling circle model, including the production of genome concatemers that are cleaved into monomers and packed into pre-assembled capsids [29,34]. To facilitate this cycle of generating virus progeny, HSV-1 also hijacks cellular components. One example of a DDR pathway that positively influences productive growth of HSV-1, and leads to impaired virus replication when absent, is the Fanconi anemia (FA) pathway [30,35]. Activation of the FA pathway after HSV-1 infection is initiated by DNA polymerase through mono-ubiquitination of the Fanconi Anemia Complementation Group I and Fanconi anemia group D2 protein (FANCI/FANCD2) complex. The FANCD2 protein then travels to the replication compartments in the nucleus and interacts directly with the HSV-1 single-stranded DNA binding protein ICP8 to potently stimulate viral replication. Interestingly, HSV-1 activates the FA pathway, possibly to suppress antiviral functions of c-NHEJ [35]. However, further investigations into the interaction of HSV-1 with NHEJ proteins by Muylaert and colleagues found that the inhibition of LIG4 and XRCC4 by RNA interference (RNAi) reduced HSV-1 virus production and interfered with viral DNA synthesis in human fibroblasts [36]. Besides NHEJ-proteins, many other DDR factors have been found to interact with HSV-1 and only by dissecting each of these factors will we receive a full picture of the HSV-life cycle. Interesting examples are Ataxia-telangiectasia-mutated protein (ATM) and Ataxia-telangiectasia and Rad3-related protein (ATR), which have functions connected to HR and, like DNA-PKcs, are members of the Phosphatidylinositol 3-kinase-related kinases (PIKK) family [37]. ATM, ATR, and DNA-PKcs are all tightly regulated through specific co-factors, share similar organisational and structural features, and in some cases regulate one another [38]. While DNA-PKcs has antiviral properties in HSV-1 infection, the virus uses ATR and ATM for its own advantage to promote efficient replication. HSV-1 also inhibits downstream DDR signaling via ATR, highlighting the often conflicting nature of viral interactions [39,40,41,42,43].

To fully understand the interaction of virus and cell, it is crucial to uncover all factors involved in the interplay. Here we present data indicating that the NHEJ protein PAXX, which shares structural similarities with XRCC4 and XLF but is unstudied in the context of virus infection, acts to restrict HSV-1 in a manner that is different from those mediated by other DDR proteins.

## 2. Materials and Methods

### 2.1. Cell Culture

All cells were incubated at 37 °C, 5% CO_2_, and 3% O_2_. U2OS (human osteosarcoma) cells and Vero cells were grown in Dulbecco’s Modified Eagle Medium (DMEM; Gibco, Waltham, MA, USA) with 10% volume per volume (*v*/*v*) heat-inactivated foetal bovine serum (FBS; Sera Laboratories International Ltd., West Sussex, UK) and 50 μg/mL of penicillin/streptomycin (P/S; Gibco, Fisher Scientific UK Ltd., Loughborough, UK). Retinal pigment epithelia (RPE-1) cells were cultured in DMEM-F12 with Glutamax, 10% *v*/*v* FBS, 0.2% weight/volume (*w*/*v*) sodium bicarbonate, and 50 μg/mL P/S. Mouse embryonic fibroblasts (MEFs) were maintained in DMEM-F12 with Glutamax (Gibco), 15% *v*/*v* FBS, 0.001% *v*/*v* 2-mercaptoethanol, non-essential amino acids (glycine, l-alanine, l-asparagine, l-aspartic acid, l-glutamic acid, l-proline, l-serine; all 100 nM), 1 mM sodium pyruvate, and 50 μg/mL P/S.

### 2.2. Transfection

TransIT-LT1 (Mirus Bio LLC, Madison, WI, USA) was used for plasmid DNA transfection. For optimum transfection efficiency cells were seeded at 70–80% confluency. TransIT-LT1 was mixed with OptiMEM (Gibco) at a ratio of 1:33 and incubated at room temperature for 5 min, DNA was then added at a ratio of 1 μg DNA:3 μL TransIT-LT1, and incubated for a minimum of 15 min. The transfection mix was added to the cells directly into the culture medium.

### 2.3. Viruses

Strain 17+ (S17) HSV-1 virus was used as the wild-type (WT) strain and was a kind gift from Professor Stacey Efstathiou. An S17 virus lacking ICP0 (referred to in the literature as *dl1403*) was a kind gift from Professor Gill Elliot [44]. *dl1403* contains a 2 kb deletion within the TR_L_ and IR_L_ copies of *Vmw110* (the gene encoding the immediate-early protein ICP0) which encode 105 amino acids from the original N-terminus followed by 56 amino acids altered by a frame-shift. The ΔgE/Viral protein 26 (VP26)-yellow fluorescent protein (YFP) virus is derived from a strain-16 (S16) parental virus and was a gift from Dr. Colin Crump.

### 2.4. Plaque Assay Titration of HSV-1

Vero cells, or U20S cells for *dl1403*, were seeded into 6-well plates to be confluent at the time of titration. The virus stock was serially diluted 10-fold in DMEM plus 2.5% *v*/*v* FBS, and applied to the cells. The plates were rocked every 15 min for one hour, and then the medium was replaced with 1.5% carboxymethyl cellulose (CMC) complemented with a final concentration of 1× minimum essential medium (MEM). The plates were incubated at 37 °C, 5% CO_2_ and 3% O_2_ until plaques were observed. The semi-solid overlay was then removed and cells were fixed and stained with 5% (*v*/*v*) crystal violet (Sigma, St. Louis, MO, USA) and 25% (*v*/*v*) ethanol for one hour, washed with water, and the plaques counted. Each experiment was conducted three times with three biological replicates.

### 2.5. Virus Titre Assay

MEFs were seeded into 6-well plates, and RPE-1 cells were seeded into T25 flasks. Prior to infection, cells were counted and infected at multiplicity of infection (MOI) of 0.01 or 4. At the desired time post-infection, cells were scraped into the growth medium. The cell suspensions of all samples were freeze-thawed three times, and stored at −80 °C. Infectious virion numbers were determined by titration of each sample in duplicate on Vero cells. Each experiment was conducted at least three times with three biological replicates.

### 2.6. Cellular RNA Extraction

Cells were lysed in situ using 250 μL of lysis buffer containing 4 M guanidine thiocyanate, 25 mM Tris pH 7, and 143 mM 2-mercaptoethanol. Consequently 250 μL of ethanol was added, and the solution was applied to a silica column (Epoch Life Science, Inc., Sugar Land, TX, USA) and centrifuged; all centrifugation steps were performed for 30 s at 16,000× *g*. The bound RNA was washed by centrifugation with 500 μL of buffer containing 1 M guanidine thiocyanate, 25 mM Tris pH 7, and 10% ethanol, followed by a second washing step with 500 μL of wash buffer 2 (25 mM Tris pH 7 and 70% (*v*/*v*) ethanol). An additional wash step was performed with 500 μL of Wash Buffer 2 and centrifuged for two minutes at 16,000× *g*. The RNA was then eluted by centrifugation in 30 μL of nuclease-free water. The RNA concentration was determined using a NanoDrop 2000 Spectrophotometer (Thermo Scientific, Waltham, MA, USA).

### 2.7. cDNA Synthesis

Complementary DNA (cDNA) was generated from 500 ng of isolated RNA. 1 µL of a 10 mM deoxynucleotide triphosphate (dNTP) mixture and 500 ng of oligo(dT) (both Thermo Scientific, Waltham, MA, USA) were incubated with the RNA at 65 °C for five minutes in a total volume of 13 µL. 40 U of RNaseOUT recombinant RNase inhibitor (Invitrogen, Carlsbad, CA, USA), 50 U of Superscript III Reverse Transcriptase (Invitrogen), 2 µL of 10× first strand buffer, and 1 μL of 0.1 M dithiothreitol (DTT) were then added and the volume adjusted to 20 µL with nuclease-free water. This solution was incubated for one hour at 50 °C, and then at 72 °C for 15 min.

### 2.8. Quantitative Real Time-Polymerase Chain Reaction (qPCR)

cDNA was diluted 1:3 in nuclease-free water and 2 μL was added into wells of a MicroAmp Fast Optical 96-well or 384-well reaction plate (Applied Biosystems, Foster City, CA, USA). A mastermix containing 5 μL Fast SYBR Green Master Mix (Life Technologies, Carlsbad, CA, USA) and 1 μL of 10 mM stocks of each of a forward and reverse primer per well were added per well (Table 1). The plate was centrifuged for one minute at 180× *g* before being analysed on a 7500 Fast Real-Time PCR System (Applied Biosystems). Each experiment was conducted at least three times with three biological replicates.

### 2.9. Isolation of Cellular and Viral DNA for Quantification by qPCR

Infected cells were scraped into growth medium, centrifuged for five minutes at 400× *g*, and the resulting pellet was resuspended in lysis buffer (10× the volume of the pellet) consisting of 5 μM sodium dodecyl sulfate (SDS), 10 mM Tris-HCl pH 8.3, and 100 μg/mL proteinase K. The cells and buffer were then incubated at 50 °C for 90 min, before proteinase K was heat inactivated at 95 °C for 10 min.

### 2.10. Quantification of Viral DNA by qPCR

For qPCR of cellular and viral genomes, primers and probes, tagged with the fluorophores cyanine5 (Cy5) and 6-carboxyfluorescein (FAM), coupled with a BlackBerry quencher (BBQ), targeting the promoter regions of human glyceraldehyde 3-phosphate dehydrogenase (GAPDH) and HSV-1 ICP0 were used (Table 2). A mastermix was created with final concentrations of 8 mM MgCl2 (Qiagen, Hilden, Germany), 0.8 mM dNTPs (Thermo Scientific), 5% (*v*/*v*) dimethyl sulfoxide (DMSO), 0.8 U of HotStarTaq DNA polymerase (Qiagen), and Taq polymerase buffer. Primers and probes (TIB Molbiol, Berlin, Germany) were added as detailed in Table 2. 19 μL of this mastermix and 1 μL of the sample were mixed (all samples were analysed in triplicates). A standard curve was generated by using a dilution series of two plasmids each containing one of the promoter regions. qPCR of the samples and standards was carried out simultaneously on a Rotorgene 3000 (Corbett Research, Hilly St, Australia). The reaction was held at 95 °C for 15 min, before 45 cycles of 95 °C for 30 s, and 60 °C for 60 s. The standard-adjusted signal from the ICP0 reaction was divided by the standard-adjusted signal for GAPDH to give a relative signal intensity. Each experiment was conducted at least three times with three biological replicates.

### 2.11. Isolation of Viral DNA for Southern Blotting

Infected cells were scraped into the growth medium, and centrifuged at 400× *g* for five minutes. The pellet was resuspended in 1.6 mL of buffer containing 10 mM Tris, 50 mM Ethylenediaminetetraacetic acid (EDTA), 0.5% (*w*/*v*) SDS and 163 μL/mL proteinase K before incubation at 37 °C overnight. The next day, an equal volume of phenol was added and gently mixed before the solution was centrifuged for 10 min at 1600× *g* and 20 °C. The top, aqueous layer was transferred to a clean tube, and the phenol wash was repeated. An equal volume of chloroform was added to the aqueous layer, and the solution was centrifuged for 10 min at 1600× *g* and 20 °C. The aqueous layer was isolated and NaCl was added to a final concentration of 0.2 M. Absolute ethanol was added to a final concentration of 29% (*v*/*v*), and the solution was mixed. The sample was then kept at −20 °C overnight before 15 min of centrifugation at 2900× *g* and 4 °C. The supernatant was removed and the precipitated DNA pellet was air-dried, before resuspension in 300 μL of 10 mM Tris and 1 mM EDTA. The concentration of the samples was measured with a NanoDrop 2000 Spectrophotometer.

### 2.12. Creation of Hybridisation Probe for Southern Blotting

The HSV-1 BamHI K fragment (nucleotides 123459 to 129403 of strain S17 [45], were excised from a donor plasmid (pAT153 containing fragment Kpn A), gel-purified, and 100 ng of the excised fragment were added to nuclease-free water to a total volume of 34 µL. After boiling for five minutes, the solution was placed on ice for five minutes. A dNTP mix was created with final concentrations of 0.17 mM biotin-14-dATP (Invitrogen), 0.17 mM biotin-14-dCTP (Invitrogen), and 0.98 mM of each of dATP, dTTP, dGTP, and dCTP. The BamHI K fragment was then added to 5 μL of the dNTP mix, 1 μL of Klenow enzyme (NEB, Ipswich, MA, USA), and 10 μL of 5× labelling mix (NEB) and incubated at 37 °C for three hours. 200 μg of salmon sperm DNA and 30 μL of water were added prior to purification using a Qiagen PCR purification kit.

### 2.13. Southern Blotting

Prior to Southern blotting, isolated DNA was digested with BamHI. The samples were then analysed on a NanoDrop 2000 Spectrophotometer, and an equal mass of DNA from each sample was loaded onto a 0.8% (*w*/*v*) agarose gel containing ethidium bromide along with biotinylated molecular weight markers (NEB). The gel was run at 16 V overnight, and once sufficient separation of the samples was observed, the gel was rocked in 0.25 M HCl for 20 min to hydrolyse the DNA. The gel was then rocked in 0.4 M NaOH for 30 min and the transfer of separated samples on a membrane was conducted using a semi-dry transfer overnight.

Filter paper was washed in 4× standard saline phosphate EDTA (SSPE, pH 7.4; 750 mM NaCl, 800 mM NaH_2_PO_4_·H_2_O, and 20 mM Na_2_EDTA·2H_2_O) for 30 min. The filter paper was blocked in prehybridisation buffer (final concentrations of 1× Denhardt’s solution, 200 μg/mL boiled salmon sperm DNA, 4× SSPE, 2% (*w*/*v*) SDS, and 10% (*w*/*v*) dextran sulphate) at 65 °C in a hybridisation oven for a two hours. The probe was then added to the prehybridisation buffer and allowed to hybridise at 65 °C overnight. The filter paper was washed twice in each of three wash buffers (2× SSPE; 2× SSPE, 1% (*w*/*v*) SDS; 0.1× SSPE) for 15 min at 65 °C. The filter paper was then incubated at room temperature in Odyssey block (LI-COR Biosciences, Lincoln, NE, USA) containing 1.2% (*w*/*v*) SDS for 30 min, followed by a 30 min incubation in Odyssey block, 1.2% (*w*/*v*) SDS, and Streptavidin-IRDye 800 CW (LI-COR Biosciences). The filter paper was washed in phosphate-buffered saline with Tween-20 (PBST) three times, and visualised using a LI-COR imaging system. Each experiment was conducted at least three times with three biological replicates.

### 2.14. Immunofluorescence

Cells were seeded onto sterile 13 mm coverslips in standard growth medium to be 60–70% confluent at the time of fixation. Following treatment cells were fixed by aspirating the medium and then incubating in 250 mM pH 7.4 4-(2-hydroxyethyl)-1-piperazineethanesulfonic acid (HEPES) with 4% (*w*/*v*) paraformaldehyde (PFA) at 4 °C, and then a further 10 min in 8% PFA and 250 mM pH 7.4 HEPES. The coverslips were washed twice in PBS, permeabilised in 1% (*v*/*v*) Triton X-100 in PBS for five minutes, and washed twice more in PBS. 5% (*w*/*v*) milk from powder (Premier Food Groups) in PBS was used as a blocking agent for one hour at room temperature on a rocking platform. The primary antibody (anti-PAXX, Sigma) was diluted 1:1000 in 1% (*w*/*v*) milk in PBS, and 50 μL of the dilution was pipetted onto a sheet of Parafilm M (Bemis, Neenah, WI, USA). The coverslips were placed sample-side down onto the antibodies, and incubated at room temperature in a humidified chamber in the dark for one hour. Following this incubation, the coverslips were washed three times in PBS, and then incubated for 30 min in 200 μL of fluorophore-conjugated secondary antibodies (anti-rabbit or anti-mouse conjugated to Alexa Fluor488 or Alexa Fluor546; Invitrogen) diluted 1:1000 in 1% *w*/*v* milk. The plates were washed twice in PBS and once in water. Coverslips were mounted onto slides using 10 μL of mounting solution (25% glycerol (*v*/*v*), 0.1 M Tris pH 8.5, 10% Mowiol 4-88 *w*/*v* containing 4′, 6-diamidino-2-phenylindole (DAPI)) and allowed to set in the dark overnight. Samples were visualised and imaged using a Zeiss Pascal Confocal Microscope (LSM 800, Carl Zeiss Ltd., Cambridge, UK) at 63× magnification under oil, and images were collected using Zeiss LSM Image Browser (ZEN lite 2012 software, Carl Zeiss Ltd., Cambridge, UK). Each experiment was conducted at least three times with three biological replicates.

### 2.15. Immunoblotting

Cells were scraped into the medium, and centrifuged at 1900× *g* for five minutes. The supernatant was aspirated, and the pellet resuspended in PBS before centrifuging at 1900× *g* for five minutes. The supernatant was aspirated, and the cells were lysed in 250 μL of lysis buffer (150 mM NaCl, 20 mM Tris-HCl (pH 7.4), 10 mM CaCl_2_, 0.1% (*v*/*v*) Triton X-100, and 10% (*v*/*v*) glycerol) on ice for 30 min. After the 30 min the cell lysate was centrifuged at 16,000× *g* at 4 °C for 10 min. The supernatant was flash-frozen in liquid nitrogen and stored at −80 °C. Prior to loading onto a gel, a bicinchoninic acid (BCA) assay (Thermo Scientific) was used to determine protein concentrations, allowing for the equal loading of protein samples. 6× sample loading buffer (300 mM Tris-HCl (pH 6.8), 12% (*w*/*v*) SDS, 60% (*v*/*v*) glycerol, 0.6% (*w*/*v*) bromophenol blue, and 600 μM β-mercaptoethanol (BME)) was added to the samples before heating at 94 °C for five minutes prior to electrophoresis. Following SDS-polyacrylamide gel electrophoresis (SDS-PAGE), gels, nitrocellulose membranes (GE Healthcare, Little Chalfont, UK) and blot paper (BioRad, Hercules, CA, USA) were equilibrated in transfer buffer (20% (*v*/*v*) methanol, 2.5 mM Tris Base, and 19.2 mM glycine) for five minutes. A Trans-Blot Turbo semi-dry transfer machine (BioRad) was used for protein transfer onto the membrane at 25 V for 30 min. After transfer, the nitrocellulose membrane was blocked in 5% (*w*/*v*) milk for one hour. The membrane was washed three times in PBS with 0.025% (*v*/*v*) Tween 20 (PBST), and incubated overnight at 4 °C in primary antibody diluted in PBST (Table 3). After three washes in PBST the membrane was incubated for one hour in 5% (*w*/*v*) milk containing the secondary antibody (Table 3). The membrane was washed three times in PBST, air-dried in the dark, and visualised using a LI-COR imaging system, or with enhanced chemiluminescent (ECL) reagent (125 mM luminol, 0.4 mM curaric acid, 0.1 M Tris pH 8.8), using a developing film processor (SRX101A, Konica Minolta, Tokyo, Japan). Each experiment was conducted at least three times with three biological replicates.

## 3. Results

### 3.1. HSV-1 Infection Induces Changes in PAXX Distribution

**Figure 2:**
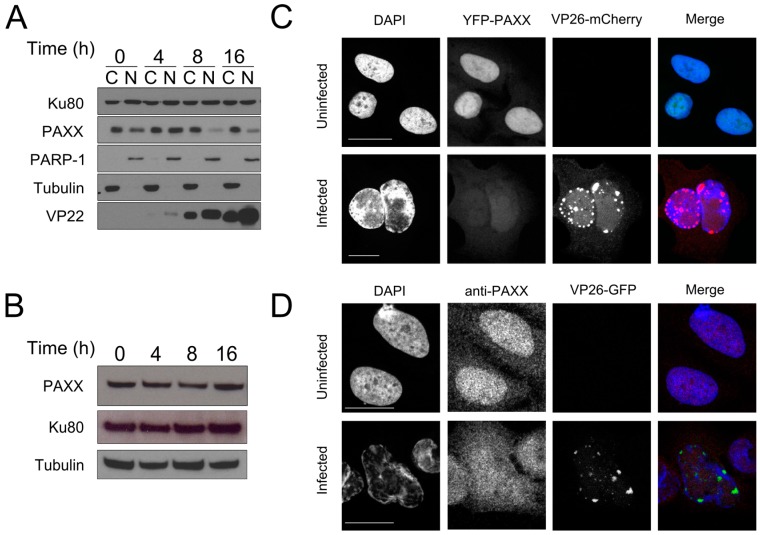
PAXX distribution changes during HSV-1 infection. (**A**) U2OS cells were infected at a multiplicity of infection (MOI) of 10. Cells were harvested and the cytoplasmic (C) and nuclear (N) fractions separated and probed by immunoblotting for PAXX. Poly (ADP-Ribose)-Polymerase 1 (PARP-1) and tubulin controlled for fractionation, VP22 demonstrated successful infection, and Ku80 was used as a loading control; (**B**) U2OS cells were infected at MOI 10 and whole cell lysates immunoblotted with the indicated antibodies; (**C**) Yellow fluorescent protein (YFP)-PAXX was ectopically expressed in U2OS cells, and these were infected with HSV-1 VP26-mCherry at MOI 1. 16 h after infection slides were imaged by confocal microscopy; (**D**) U2OS cells were infected with ΔgE VP26-YFP HSV-1 at an MOI of 1 for 16 h and analysed by immunofluorescence. Scale bars in (**C**,**D**) denote 20 μm.

The HSV-1 protein ICP0 induces degradation of DNA-PKcs, a component of the c-NHEJ machinery that is required for sensing of viral DNA [16,17,46]. Since PAXX and DNA-PKcs are both part of the c-NHEJ machinery [6], we hypothesised that PAXX may also be degraded during HSV-1 infection. The HSV-1 genome replicates in the nucleus and PAXX has been reported to be predominantly nuclear [6], so we analysed the effect of HSV-1 infection on nuclear PAXX. U2OS cells were infected with S17+ HSV-1 at an MOI of 10 and the cells were lysed at various times after infection. Samples were fractionated into nuclear and cytoplasmic compartments, and immunoblotting was performed to visualise PAXX levels in these compartments during infection (Figure 2A). We observed that prior to infection PAXX is both nuclear and cytoplasmic but at both 8 and 16 h post-infection a large reduction in levels of nuclear PAXX was observed. Cytoplasmic levels of PAXX, however, remained largely unchanged. Despite this specific reduction in nuclear PAXX, immunoblotting of whole cell lysates indicated that overall PAXX levels were not significantly reduced during infection (Figure 2B).

These findings were complemented using confocal microscopy studies in which YFP-tagged PAXX was ectopically expressed in U2OS cells, and in the absence of infection had both nuclear and cytoplasmic localisation (Figure 2C). Following HSV-1 infection, however, PAXX was only weakly observed in the nucleus, but cytoplasmic levels remained unchanged. Immunofluorescence of endogenous protein during HSV-1 infection is limited by the presence of the HSV-1 IgG Fc receptor that can bind non-specifically to the primary and/or secondary antibodies used in these assays. In the absence of the HSV-1 glycoprotein gE this complex cannot form [47], so to visualise endogenous PAXX during infection U2OS cells were infected with a ΔgE YFP-VP26 HSV-1 virus (Figure 2D). This experiment confirmed the observations made using ectopic PAXX expression, and together the data showed that nuclear PAXX protein is depleted early in HSV-1 infection. The above observation, that in uninfected cells PAXX is predominantly localised in the nucleus whereas during infection PAXX is dispersed uniformly in the cell was also confirmed in RPE cells (Supplementary Figure S1).

### 3.2. Paxx^−/−^ MEFs Are Not Defective in Type I Interferon Production during HSV-1 Infection

**Figure 3:**
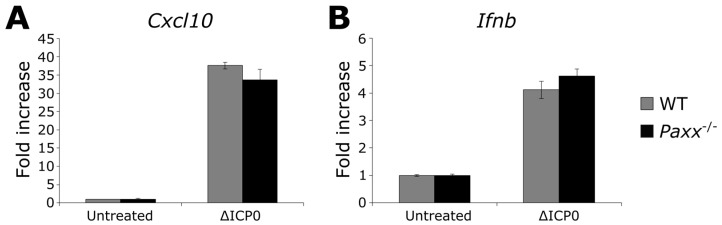
PAXX does not affect innate immune responses to HSV-1 ΔICP0 infection. WT or *Paxx*^−/−^ Mouse embryo fibroblasts (MEFs) were infected with MOI 5 ΔICP0 for 5 hr, and qPCR was used to analyse the transcription of (**A**) *Cxcl10*; and (**B**) *Ifnb. n* = 3. Error bars denote mean +/− standard error of the mean (SEM).

Several DDR proteins, including some c-NHEJ components, are involved in the intracellular innate immune sensing of HSV-1 DNA that is essential for type-I interferon production during infection [14,16,17,18,19,27]. In response, the virus has evolved mechanisms to inactivate such proteins to enhance its replication [31,46,48,49,50]. Since HSV-1 infection results in the loss of nuclear PAXX, we considered the possibility that PAXX is also involved in the innate sensing of viral DNA during HSV-1 infection. To test this we used passage 1 MEFs generated from wild-type (WT) and *Paxx*^−/−^ mice [12]. Early passage MEFs respond to intracellular DNA stimulation by producing significant amounts of IFN-I, chemokines, and cytokines [16,51]. WT and *Paxx*^−/−^ MEFs were infected at an MOI of 5 with ΔICP0 HSV-1. ICP0 is a potent inhibitor of innate immune-signaling pathways so use of this deletion virus enhances IFN-I production from infected cells [49,52,53]. RNA was harvested after six hours of infection, and cDNA was created for analysis by qPCR. No defect in *Ifnb* or *Cxcl10* transcription was observed in the *Paxx*^−/−^ MEFs (Figure 3). As a result, we rejected the hypothesis that PAXX is required for the production of IFN-I and cytokines in response to HSV-1 infection.

### 3.3. PAXX Is Not Required to Create Endless Forms of the HSV-1 Genome

**Figure 4:**
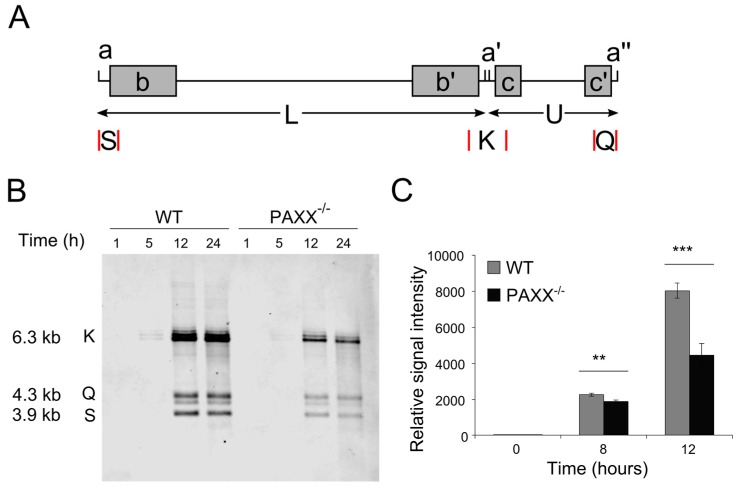
PAXX does not affect the proportion of endless HSV-1 genomes, but promotes genome replication. (**A**) Linear structure of the HSV-1 genome, indicating repetitive elements (a, a′, a″) and the S, K, and Q fragments created by cleavage with BamHI (selected restriction sites are denoted by red lines); (**B**) WT and *PAXX*^−/−^ cells were infected at MOI 4, and total cellular and viral DNA was isolated. The HSV-1 Q, K, and S fragments were visualised by southern blot following digestion with BamHI; (**C**) WT and *PAXX*^−/−^ RPE-1 cells were infected at an MOI of 4, and genome copy numbers were quantified using TaqMan qPCR. *n* = 3. Statistical significance, calculated by Student’s *t*-test, is denoted by stars—** = *p* < 0.02, *** = *p* < 0.01. *n* = 3. Error bars denote mean +/− SEM.

As PAXX is not required for HSV-1-induced IFN-I production, we considered alternative roles for PAXX in HSV-1 infection. During infection, circular and concatemeric forms of the HSV-1 genome are formed as endless replication intermediates, and some DDR proteins negatively affect production of these endless species by interfering with their production [36,54]. To test whether PAXX affects the formation of endless genome structures or their subsequent processing, WT and *PAXX*^−/−^ human retinal pigment epithelial (RPE-1) cells [6] were infected at MOI 4, and at various times after infection viral and cellular DNA was isolated. BamHI restriction enzyme digestion was used to create, amongst others, tHSV-1 genome fragments K, Q, and S (Figure 4A). These fragments are indicative of HSV-1 genome structure because K fragments are formed where the terminal Q and S fragments are combined [36]; therefore samples with higher levels of endless genomes will have a higher proportion of K fragments than those with monomeric genomes. The K, Q, and S fragments can be visualised on a Southern blot using biotinylated, randomly-primed, single-stranded DNA (ssDNA) probes recognising the K fragment, and streptavidin conjugated to fluorescent markers. After five hours of infection endless forms of the genome were produced in cells of both genotypes (Figure 4B). After 12 and 24 h of infection monomeric forms (represented by Q and S) were visible in both WT and *PAXX*^−/−^ cells, and the relative levels of K to Q and S fragments was similar between genotypes. It has been suggested that cleavage of the HSV-1 genome occurs simultaneously with its packaging into the capsid [55,56], and so the presence of the Q and S fragments after 12 and 24 h suggested that packaging is occurring efficiently in the presence or absence of PAXX. Interestingly, overall viral DNA levels were lower in *PAXX*^−/−^ cells than in WT cells, implying that PAXX promotes HSV-1 genome replication.

### 3.4. PAXX^−/−^ Cells Produce Fewer Viral Genomes than WT Cells

Many DDR proteins promote replication of the HSV-1 genome [36,42,57,58,59]. To follow up on our observation that *PAXX*^−/−^ cells produced less viral DNA than their WT counterparts, we quantified viral genome production directly by using qPCR. WT and *PAXX*^−/−^ RPE-1 cells were infected at MOI 4 with HSV-1, and viral and cellular DNA was isolated at various times post-infection. Primers and probes recognising the promoter regions of *ICP0* and *GAPDH* were used so that only genomic DNA, and not mRNA, would be amplified. After eight and 12 h of infection, there were significantly more genomes per cell in WT RPE-1 cells than in *PAXX*^−/−^ cells (Figure 4C). These data supported the observations made with southern blotting (Figure 4B), and suggested that PAXX may contribute to creating an environment that promotes HSV-1 genome replication.

### 3.5. Viral Gene Expression and Protein Production Are Unaffected by PAXX

**Figure 5:**
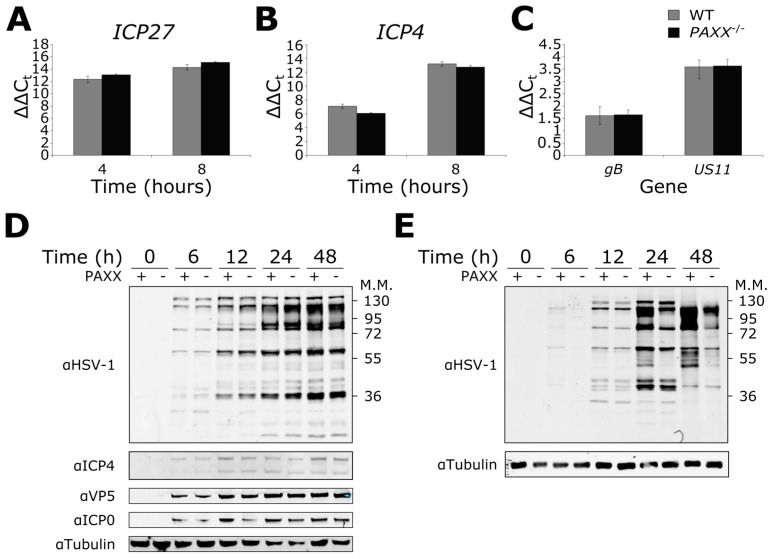
Effects of PAXX on viral gene transcription and protein production. Wild type (WT) or *PAXX*^−/−^ RPE-1 cells were infected at an MOI of 5, and RNA extracted. qPCR was used to quantify viral transcription of (**A**) *ICP27*; (**B**) *ICP4*; *(***C**) *gB* and *US11*; (**D**) RPE-1 cells were infected at MOI 1 and harvested for protein at various times post infection. An anti-HSV-1 antibody was used, along with specific antibodies. (**E**) As in (**D**), but in MEFs. Error bars denote mean +/− SEM. *n* = 3.

The expression of the HSV-1 γ2 true late genes is dependent on viral genome replication [60,61]. We therefore hypothesised that HSV-1 infection in *PAXX*^−/−^ cells might result in altered late gene expression because genome replication is impaired in *PAXX*^−/−^ cells. To examine this possibility, WT and *PAXX*^−/−^ cells were infected at MOI 5, and RNA was isolated after four or eight hours of infection. These time points were selected to investigate immediate-early gene expression, and because southern blotting and qPCR (Section 3.3 and Section 3.4) had demonstrated that DNA replication had started by six hours and so late gene expression should also be observed. We used qPCR to analyse the expression of immediate-early (*ICP27* and *ICP4*), late (*gB*) and true late (*US11*) genes. Perhaps unexpectedly, the expression of all genes analysed was similar between genotypes (Figure 5). These data suggested that PAXX does not affect the transcription of these viral genes.

To complement the observations made with gene transcription, we also investigated the effect of PAXX on HSV-1 protein expression during infection. WT and *PAXX*^−/−^ RPE-1 cells were infected at MOI 1, and protein was harvested at various times post-infection. Whole cell lysates were then immunoblotted for the immediate early proteins ICP4 and ICP0, and the γ2 true late protein VP5. Total protein levels were similar between genotypes for all gene products analysed (Figure 5D). In addition, samples were immunoblotted using an antibody raised against HSV-1 virions, which is thought to primarily recognise late gene products in the capsid. Again, there were no differences observed in the viral protein levels between the WT and *PAXX*^−/−^ cells (Figure 5D). The experiment was also carried out in MEFs; in contrast to the observations in RPE-1 cells there was slightly higher expression of some viral proteins in WT MEF cells observed with the anti-HSV-1 antibody as early as six hours post-infection (Figure 5E). This became more marked after 24 and 48 h of infection, although not all proteins were affected. Together these data suggested that viral transcription in RPE-1 cells is unaffected by PAXX, but that PAXX may play some role in regulating viral gene expression in MEFs. In addition whole cell lysates from RPE-1 WT and *PAXX*^−/−^ cells infected with a high MOI (MOI 4) were immunoblotted using an anti-HSV-1 antibody and showed no difference in protein expression between the two cell types (Supplementary Figure S2). A high MOI infection experiment was omitted in MEFs, as infecting them at MOI 4 led to rapid cell detachment and death.

### 3.6. PAXX^−/−^ Cells Produce More Infectious Virions than WT Cells

**Figure 6:**
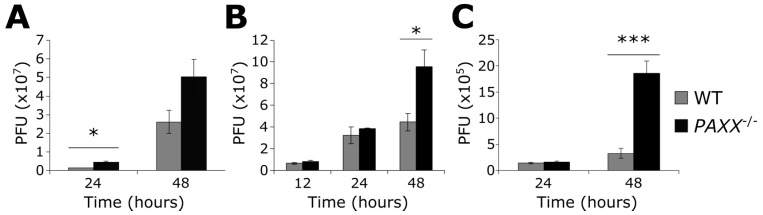
PAXX restricts production of infectious HSV-1 virions. WT or *PAXX*^−/−^ RPE-1 cells were infected with HSV-1 at MOI (**A**) 0.01, or (**B**) 4, and infectious virions were titrated onto vero cells to determine virus titre (plaque-forming units; PFU); (**C**) WT or *Paxx*^−/−^ MEFs were infected with HSV-1 at an MOI of 0.01, and infectious virions were titrated onto vero cells. Statistical significance, calculated by Student’s *t*-test, is denoted by stars—* = *p* < 0.05, *** = *p* < 0.01. *n* = 3. Error bars denote mean +/− SEM.

The observations that there are fewer viral genomes in *PAXX*^−/−^ cells than in WT cells, and that PAXX may promote viral protein production suggested that PAXX might alter the production of infectious HSV-1 virions. To test this hypothesis, WT and *PAXX*^−/−^ RPE-1 cells were infected at an MOI of 0.01 or 4 with HSV-1, and at various times after infection both cell-associated virions and virions free in the growth medium were titrated together onto vero cells to assess viral titres. *PAXX*^−/−^ cells consistently produced more infectious virions than WT cells across various time points and at both high and low MOIs (Figure 6A,B). *Paxx*^−/−^ MEFs also produced significantly more infectious virions than WT cells after 48 h of infection at MOI 0.01 (Figure 6C). These data thereby established that PAXX acts to restrict the production of infectious virus in both human and murine cells.

## 4. Discussion

PAXX acts to restrict HSV-1 infection by reducing the efficiency by which infectious virions are formed in cells. In the presence of PAXX, there are increased levels of viral DNA and some viral proteins, but a reduced amount of infectious virus particles compared with cells that lack PAXX. This increase in virus production in the absence of PAXX is not due to gross changes in viral genome structure or related to viral gene expression. It is not yet clear how PAXX acts to alter the efficiency of HSV-1 infection. PAXX possibly interacts with a so far unknown target, sequestering the viral DNA in the cells in a way which prevents the release of fully infectious virus particles. In contrast, further upstream in the viral replication pathway, PAXX might have a supportive role in the HSV-1 life cycle in a similar manner to its role as a stabiliser in c-NHEJ. Our observations are in keeping with the growing literature on the important function of DDR proteins in regulating multiple aspects of virus infection.

A number of cellular DDR proteins either positively or negatively affect HSV-1. The mechanisms can generally be divided into those affecting HSV-1 genome replication and transcription [30], and those which are linked to the innate immune response [27]. Efficient replication of the HSV-1 genome is limited in the absence of host factors such as ATM, MRN, or WRN [57,58,62], indicating that such factors promote productive infection. By contrast, DDR proteins such as DNA-PKcs inhibit replication, probably through recognition of nicks and gaps in the dsDNA HSV-1 genome [28]. In an attempt to explain these observations, specific DDR pathways can be described as beneficial or detrimental for HSV-1 infection, but they can also have more complex roles. Indeed, although the HSV-1 protein UL12 activates the single-strand annealing (SSA) pathway of DNA DSB repair, it decreases rates of HR and c-NHEJ [63]. This is despite the fact that many HR proteins are required for efficient HSV-1 production [57,58,62]. The c-NHEJ pathway, which includes PAXX, is another good example of a pathway with both beneficial and detrimental components in the context of HSV-1. DNA LIG4 and XRCC4 promote HSV-1 replication [36], while DNA-PKcs and the Ku heterodimer restrict infection [28,46,58]. Furthermore, we have shown that the impact of PAXX is different from that of other c-NHEJ proteins, including LIG4 and XRCC4, despite the structural homology between PAXX and XRCC4 [6]. Although PAXX, LIG4, and XRCC4 all support viral genome replication, knockdown of LIG4 or XRCC4 reduces genome circularisation and reduces the output of infectious virus [36], whereas knocking out PAXX has no discernible effect on the formation of endless genomes, but instead increased infectious virus production. These data imply that PAXX plays a hitherto undocumented role during HSV-1 infection.

PAXX is not the only DDR protein shown to have both beneficial and restrictive functions during HSV-1 infection; ATR is recruited to viral replication centres and promotes viral gene expression and production of infectious virus, yet the signaling downstream of ATR is inhibited by HSV-1 [39,64]. It therefore seems possible that LIG4 and XRCC4 regulate downstream DDR signaling differently to PAXX. HSV-1 may regulate DDR signaling pathways because they direct diverse cellular stress responses, such as the induction of innate immune responses, cell death and control of the cell cycle, all of which can affect HSV-1 infection. In many instances DDR pathways also regulate each other, and so pathways beneficial to the virus may be inhibited by other DDR pathways. HSV-1 induces the FA pathway, which may assist in the suppression of antiviral c-NHEJ functions [35] such as DNA-PKcs-mediated inhibition of the HR pathway [65]. Whether HSV-1 genome replication uses rolling circle replication or recombination to create concatemers remains controversial [30,34], but recombination is at least thought to be important in concatemer formation. Therefore the loss of PAXX might also relieve inhibition of pathways such as HR that can promote HSV-1 infection. However, we have found that there is no increase in the formation of endless HSV-1 genomes in *PAXX*^−/−^ cells (Figure 4), suggesting that PAXX-dependent regulation of the HR pathway is not responsible for reduced infectious virion production. It is, however, interesting to note that the change in PAXX distribution that we observed in Figure 2 occurs at a similar time to the first detection of endless forms of the viral genome (i.e., between 4 and 6 hours post-infection). It may therefore be that by this time PAXX has fulfilled its functions that are beneficial to HSV-1 replication, and it is removed from the nucleus to avoid its subsequent inhibitory role in HSV-1 infection.

Although recombination is thought to be important in the formation of endless HSV-1 genomes, LIG4 and XRCC4 have functions in this process [36] and it has been suggested that DNA-PKcs may also be involved [28]. We had hypothesised that PAXX might increase ligation of the viral genome, and thereby create and stabilise structures such as endless genomes that cannot be packaged, but the Q and S fragments detected by Southern blotting (Figure 4) are indicative of successful packaging of the genome into the viral capsid because cleavage is thought to occur concurrently with packaging [36,66]. Circularisation of the genome has been linked to latent infection [67,68], and so if PAXX does contribute to genome circularisation, this could also explain why WT cells produce fewer infectious virions. However, we observed comparable levels of viral transcription between WT and *PAXX*^−/−^ cells, which does not support this explanation.

The observation that viral transcription is comparable in WT and *PAXX*^−/−^ cells has other implications. DDR proteins, such as IFI16, RNF8, RNF168, and DNA-PKcs have been linked to the silencing of viral gene expression, largely by chromatinisation of the viral genome [30,69,70]. In the case of IFI16 this occurs quickly enough to inhibit early gene transcription. No differences were observed in the transcription of any viral gene tested in *PAXX*^−/−^ cells (Figure 5). With regard to true late (γ2) HSV-1 genes, such as US11, only being expressed following DNA replication [71], it is interesting that the reduced rate of viral genome replication in *PAXX*^−/−^ RPE-1 cells did not result in differences in true late viral gene expression or protein production. To our knowledge the relationship between the number of viral genomes and levels of gene expression has not been characterised previously, so we cannot say that a correlation should necessarily be observed. That said, equal levels of transcription despite discrepancy in genome copy number may indicate that a higher proportion of the genomes produced in the WT cells are not transcriptionally functional. Immunoblotting showed that in RPE-1 cells, protein production was also unaffected by PAXX, although some viral proteins were expressed to a higher level in WT MEFs.

Some DDR proteins also act as DNA sensors, stimulating an innate immune response following the detection of cytoplasmic or viral DNA (reviewed [27]). An innate immune response can also be triggered by DNA damage [72,73,74]. DNA-PKcs [16,17], IFI16 [14], Ku70 [19], and Rad50 [75] are DDR proteins involved in inducing the innate immune response to viral infection, and we hypothesised that PAXX may play a similar role. However, we did not observe reduced stimulation of IFN-β or CXCL10 transcription in response to ΔICP0 HSV-1 infection in *PAXX*^−/−^ cells.

Together, the data presented here demonstrate that PAXX restricts HSV-1 infection by reducing the production of infectious virions. It is therefore tempting to speculate that HSV-1-induced changes in the intracellular distribution of PAXX may be indicative of viral evasion of this restrictive effect of PAXX. However, we have also documented that PAXX positively contributes towards efficient HSV-1 genome replication. The roles of PAXX during HSV-1 infection that we have observed appear to be independent of changes to the formation of endless genomes, and are not linked to changes in viral gene expression. PAXX is also not required for the stimulation of IFN-I and cytokine transcription following HSV-1 infection. While further work is required to disentangle what are likely to be multiple roles for PAXX during HSV-1 infection, our observations contribute to the increasing evidence that DDR proteins are important for the regulation of multiple stages of virus infection.

## Figures and Tables

**Table 1:** Primer sequences used for quantitative real time-polymerase chain reaction (qPCR).

Gene Name	Primer Sequence
*ICP27* (fwd)	GTGCAAGATGTGCATCCACCACAACCTGCC
*ICP27* (rev)	GCCAGAATGACAAACACGAAGGATGCAATG
*ICP4* (fwd)	GACGTGCGCGTGGTGGTGCTGTACTCG
*ICP4* (rev)	GCGCACGGTGTTGACCACGATGAGCC
*US11* (fwd)	CTTCAGATGGCTTCGAGATCGTAG
*US11* (rev)	TGTTTACTTAAAAGGCGTGCCGT
*gB* (fwd)	TGTGTACATGTCCCCGTTTTACG
*gB* (rev)	GCGTAGAAGCCGTCAACCT
*Cxcl10* (fwd)	GTGGCATTCAAGGAGTACCTC
*Cxcl10* (rev)	GCCTTCGATTCTGGATTCAGACA
*Ifnb* (fwd)	ACATCCCTGAGGAGATTAAGCA
*Ifnb* (rev)	GCCAGGAGGTTCTCAACAATAG

**Table 2:** Primer and probe sequences used for qPCR of viral and cellular DNA.

Name	Sequence	Concentration
*ICP0* (forward)	GGAAAGGCGTGGGGTATAA	24 nM
*ICP0* (reverse)	AACGTAGGCGGGGCTTC	72 nM
*ICP0* probe	6FAM-TCGCATTTGCACCTCGGCAC-BBQ	50 nM
*GAPDH* (fwd)	CGGCTACTAGCGGTTTTACG	72 nM
*GAPDH* (rev)	AAGAAGATGCGGCTGACTGT	24 nM
*GAPDH* probe	Cy5-CACGTAGCTCAGGCCTCAAGACCT-BBQ	50 nM

**Table 3:** Antibodies used in this study.

Name	Source	Dilution
PAXX	Sigma, Dorset, UK (HPA045268)	1:1000
PARP-1	Abcam, Cambridge, UK (ab6079)	1:1000
Ku80	Santa Cruz, Dallas, TX, USA (sc1483)	1:500
Tubulin	Millipore, Burlington, MA, USA (05-829)	1:15,000
HSV-1 VP22	Gift from Geoffrey Smith (AGV031)	1:20,000
HSV-1 ICP4	Gift from Colin Crump	1:50
HSV-1 VP5	Gift from Colin Crump	1:500
HSV-1 ICP0	Abcam (ab6513)	1:2000
IRDye 800CW anti-mouse	Licor, Lincoln, NE, USA (926-32210)	1:10,000
IRDye 680RD anti-rabbit	Licor (926-68071)	1:10,000
Anti-mouse (HRP-conjugated)	Sigma (A4416)	1:10,000
Anti-rabbit (HRP-conjugated)	Sigma (A6154)	1:20,000
Anti-goat (HRP-conjugated)	Sigma (A5420)	1:20,000

## Supplementary Materials

The following are available online at www.mdpi.com/1999-4915/9/11/342/s1.
