# Supplementary material for: The Non-Homologous End Joining Protein PAXX Acts to Restrict HSV-1 Infection
Source: Viruses. 2017 Nov 16;9(11):342. doi: 10.3390/v9110342 (PMC5707549; doi:10.3390/v9110342)
Supplement: Supplementary file 1 [file viruses-09-00342-s001.pdf]

Supplementary materials

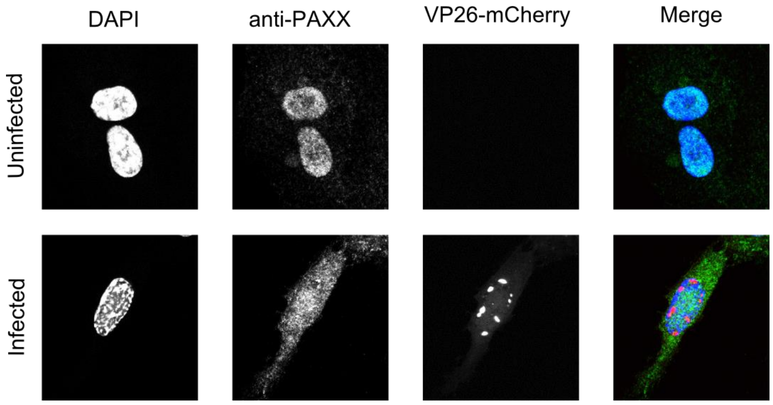

Supplementary Figure 1: PAXX localisation changes during HSV-1 infection in RPE cells. RPE cells were infected with HSV-1 VP26-GFP at an MOI of 1 for 24 hours and analysed by immunofluorescence using an anti-PAXX antibody.

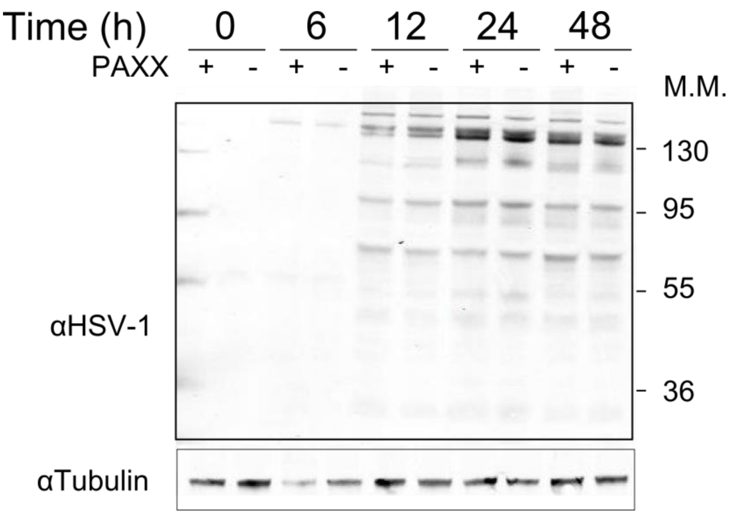

Supplementary Figure 2: Effect of PAXX on HSV-1 protein production. WT and *PAXX*$^{-/-}$ RPE-1 cells were infected at MOI 4 and analysed by immunoblotting at various time points post infection using the indicated antibodies.
